# *In vitro* modulation of MMP-2 and MMP-9 in pediatric human sarcoma cell lines by cytokines, inducers and inhibitors

**DOI:** 10.3892/ijo.2013.2159

**Published:** 2013-10-31

**Authors:** M.W. ROOMI, T. KALINOVSKY, M. RATH, A. NIEDZWIECKI

**Affiliations:** Dr Rath Research Institute, Santa Clara, CA 95050, USA

**Keywords:** matrix metalloproteinases, osteosarcoma, rhabdomyosarcoma, cytokines, inducers, inhibitors

## Abstract

The highly aggressive pediatric sarcomas are characterized by high levels of matrix metalloproteinase (MMP)-2 and MMP-9, which play crucial roles in tumor invasion and metastasis by degradation of the extracellular membrane leading to cancer cell spread to distal organs. We examined the effects of cytokines, mitogens, inducers and inhibitors on MMP-2 and -9 expression in osteosarcoma (U2OS) and rhabdomyosarcoma (RD). The selected compounds included natural cytokines and growth factors, as well as chemical compounds applied in therapy of sarcoma and natural compounds that have demonstrated anticancer therapeutic potential. These cell lines were cultured in their respective media to near confluence and the cells were washed with PBS and incubated in serum-free medium with various concentrations of several cytokines, mitogens and inhibitors. After 24 h the media were removed and analyzed for MMP-2 and -9 by gelatinase zymography and quantitated by densitometry. Osteosarcoma and rhabdomyosarcoma showed bands corresponding to MMP-2 and -9 with dose-dependent enhancement of MMP-9 with phorbol 12-myristate 13-acetate (PMA) treatment. Tumor necrosis factor-α, interleukin-1β and LPS enhanced osteosarcoma U2OS MMP-9 secretion but had no effect on MMP-2 secretion. Tumor necrosis factor-α stimulated rhabdomyosarcoma MMP-2 expression, but had no effect on MMP-9 secretion. Doxycycline, epigallocatechin gallate, nutrient mixture (NM), actinomycin-D, cyclohex-amide, retinoic acid and dexamethasone inhibited MMP-2 and -9 in U2OS osteosarcoma cells. PMA-treated RD cells showed dose-response inhibition of MMP-9 by doxycycline and epigallocatechin gallate and both MMPs by NM. Dexamethasone and actinomycin-D showed inhibition of MMP-2 secretion of RD cells. Our results show that cytokines, mitogens and inducers show variable upregulation of U2OS osteosarcoma and RD rhabdomyosarcoma MMP-2 and -9 secretion, and inhibitors demonstrate downregulation under stimulatory conditions, suggesting the application of these agents for the development of effective therapies in pediatric sarcomas.

## Introduction

Osteosarcoma, the most common bone cancer in children, usually presents in bones around the knee (80–90% in the ends of the long bones that form the knee) and secondly in the ends of the upper arm bone close to the shoulder (1). Approximately 20% of children diagnosed with osteosarcoma have an advanced stage that has metastasized to the lungs, brain and other bones (2). If metastases are present at diagnosis, 5-year survival rate is ∼15–30%, whereas the rate is closer to 40% if the cancer has spread only to the lungs or if all of the tumors can be surgically excised (3).

Rhabdomyosarcoma, the most common soft tissue sarcoma in children aged 0–14 years, comprising 50% of tumors in this age group, is a tumor of striated muscle (4). At diagnosis, roughly 50% of rhabdomyosarcoma cases consist of patients five and younger and 25% of all patients have metastatic disease (5). Of the two main histological types of pediatric rhabdomyosarcoma, embryonic and alveolar, embryonal is more prevalent, and presents in either the head and neck regions or the genitourinary tract (6). Alveolar rhabdomyosarcoma generally affects the muscles of the extremities or trunk and has been found to be more resistant to treatment and more likely to spread to regional lymph nodes than the embryonal type (7).

Numerous clinical and experimental studies have demonstrated that elevated levels of MMPs are associated with tumor growth, cancer progression, metastasis and shortened survival in patients (8.9). MMP-2 and -9 play critical roles in tumor cell invasion and metastasis by degradation of type IV collagen, a major component of the ECM and basement membrane (10–12). Secreted in their latent zymogenic form, MMP-2 (72 kDa) and MMP-9 (92 kDa) are cleaved by other MMPs or proteases to yield the activated forms of 67 kDa for MMP-2 and 64–67 kDa for MMP-9.

Environmental factors from surrounding stroma cells, ECM proteins, systemic hormones and others regulate MMP activity (10,13,14). A variety of cytokines and growth factors, such as transforming growth factor (TGF-β), hepatocyte growth factor (HGF), epidermal growth factor (EGF) and tumor necrosis factor (TNF-α) also control MMP activity (15,16). One of the most potent inducers of cancer cell proliferation is the chemical agent phorbol 12-myristate 13-aceteate (PMA). In addition, activity of MMPs is regulated at multiple levels, including transcription, modulation of messenger RNA half-life (translation), secretion, localization, activation and inhibition (17). There is little information available on the effects of various biological and chemical inducers and inhibitors in sarcomas. Among the few studies available, Rutkowski *et al* (18) investigated the correlations between serum levels of selected pro-inflammatory, hematopoietic and angiogenic cytokines and soluble cytokine receptors with the clinicopathological features and prognosis in soft tissue sarcoma patients. They found significant correlations of serum cytokine levels with tumor size and grade suggesting cytokines may be directly or indirectly involved in the progression of soft tissue sarcomas.

In this study we investigated the effects of selected cytokines, inducers, and inhibitors affecting cancer cell metabolism on the regulation of MMP-2 and -9 activities in osteosarcoma and rhabdomyosarcoma cell lines.

## Materials and methods

### Materials

Human pediatric sarcoma cell lines, osteosarcoma U2OS and rhabdomyosarcoma RD, along with their culture media were obtained from ATCC. Antibiotics, penicillin, and fetal bovine serum (FBS), were obtained from Gibco (BRL, Long Island, NY, USA). Twenty-four-well tissue culture plates were obtained from Costar (Cambrdige, MA, USA). Gelatinase zymography was performed in 10% Novex pre-cast SDS polyacrylamide gel (Invitrogen Inc.) with 0.1% gelatin in non-reducing conditions. Interleukin 1β (IL-1β), tumor necrosis factor-α (TNF-α), PMA, lipopolysaccharide (LPS), doxycycline, epigallocatechin gallate (EGCG), cyclohex-amide, actinomycin-D, retinoic acid and dexamethasone, were purchased from Sigma (St. Louis, MO, USA). The nutrient mixture (NM), prepared by VitaTech (Hayward, CA, USA) was composed of the following ingredients in the relative amounts indicated: vitamin C (as ascorbic acid and as Mg, Ca and palmitate ascorbate) 700 mg; L-lysine 1,000 mg; L-proline 750 mg; L-arginine 500 mg; N-acetyl cysteine 200 mg; standardized green tea extract (80% polyphenol) 1,000 mg; selenium 30 *μ*g; copper 2 mg; manganese 1 mg. All other reagents used were of high quality and were obtained from Sigma, unless otherwise indicated.

### Cell cultures

The sarcoma cell lines were grown in their respective media: osteosarcoma U2OS in McCoy medium and rhabdomyosarcoma in DME, supplemented with 10% FBS, penicillin (100 U/ml), and streptomycin (100 *μ*g/ml) in 24-well tissue culture plates. The cells were plated at a density of 1×10^5^ cells/ml and grown to confluency in a humidified atmosphere at 5% CO_2_ at 37°C. Serum-supplemented media were removed and the cell monolayer was washed once with PBS and with the recommended serum-free media. The cells were then incubated in 0.5 ml of serum-free medium with various cytokines, mitogens, inducers and inhibitors in triplicates, as indicated: PMA (10, 25, 50 and 100 ng/ml); TNF-α (0.1, 1, 10 and 25 ng/ml); IL-β (0.1, 1, 10 and 25 ng/ml); LPS (10, 25, 50 and 100 *μ*g/ml); EGCG (10, 25, 50 and 100 *μ*M) without and with PMA; doxycycline (10, 25, 50 and 100 *μ*M) without and with PMA; NM (10, 50, 100, 500 and 1,000 *μ*g/ml) without and with PMA; retinoic acid (50 *μ*M); dexamethasone (50 *μ*M); actinomycin-D (2 and 4 *μ*g/ml); and cyclohexamide (2 and 4 *μ*g/ml). The plates were then returned to the incubator. The conditioned medium from each treatment was collected separately, pooled, and centrifuged at 4°C for 10 min at 3,000 rpm to remove cells and cell debris. The clear supernatant was collected and used for gelatinase zymography, as described below.

### Gelatinase zymography

Gelatinase zymography was utilized because of its high sensitivity to gelatinolytic enzymatic activity and ability to detect both pro and active forms of MMP-2 and -9. Upon renaturation of the enzyme, the gelatinases digest the gelatin in the gel and reveal clear bands against an intensely stained background. Gelatinase zymography was performed in 10% Novex pre-cast SDS polyacrylamide gel in the presence of 0.1% gelatin under non-reducing conditions. Culture media (20 *μ*l) were mixed with sample buffer and loaded for SDS-PAGE with tris glycine SDS buffer, as suggested by the manufacturer (Novex). Samples were not boiled before electrophoresis. Following electrophoresis the gels were washed twice in 2.5% Triton X-100 for 30 min at room temperature to remove SDS. The gels were then incubated at 37°C overnight in substrate buffer containing 50 mM Tris-HCl and 10 mM CaCl_2_ at pH 8.0 and stained with 0.5% Coomassie Blue R250 in 50% methanol and 10% glacial acetic acid for 30 min and destained. Protein standards were run concurrently and approximate molecular weights were determined by plotting the relative mobilities of known proteins. Gelatinase zymograms were scanned using CanoScan 9950F Canon scanner at 300 dpi. The intensity of the bands was evaluated using the pixel-based densitometer program Un-Scan-It, Version 5.1, 32-bit, by Silk Scientific Corp. (Orem, UT 84059, USA), at a resolution of 1 Scanner Unit (1/100 of an inch for an image that was scanned at 100 dpi).

## Results

Table I shows the quantitative densitometry results from the effects of inducers PMA, TNF-α, IL-1β and LPS on MMP-2 and -9 secretion by osteosarcoma and rhabdomyosarcoma cell lines.

### Effect of inducers PMA, TNF-α, IL-1β and LPS on MMP-2 and -9 secretion in osteosarcoma U2OS cell line

**Figure 1.**
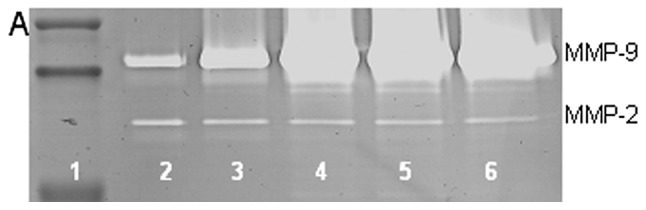

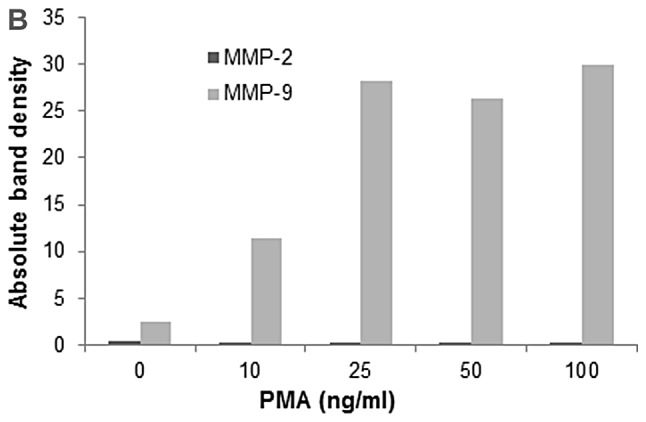
Effect of PMA on osteosarcoma U2OS MMP-2 and -9 secretions. Gelatinase zymogram (A) and densitometry analysis (B) of U2OS MMP-2 and -9 expressions. 1, Markers; 2, control; 3–6, 10, 25, 50 and 100 ng/ml PMA.

On gelatinase zymography, U2OS cells demonstrated secretion of MMP-2 and -9. PMA treatment had no significant effect on secretion of MMP-2 but stimulated MMP-9 secretion in a dose-dependent manner, with 1,200% enhancement at 100 ng/ml compared to control (linear trend R^2^=0.833), as shown in Fig. 1. TNF-α and IL-1β exerted dose-dependent stimulatory effects on MMP-9 with 3,930% enhancement at 25 ng/ml TNF-α (linear trend R^2^=0.915) and 286% enhancement at 25 ng/ml IL-1β (linear trend R^2^=0.797) and insignificant effects on MMP-2. LPS showed stimulatory effect (linear trend R^2^=0.243) on MMP-9 and up and down effects on MMP-2.

### Effect of inducers PMA, TNF-α, IL-1β and LPS on MMP-2 and -9 secretion in rhabdomyosarcoma RD cell line

**Figure 2.**
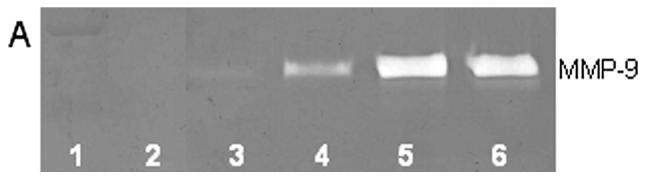

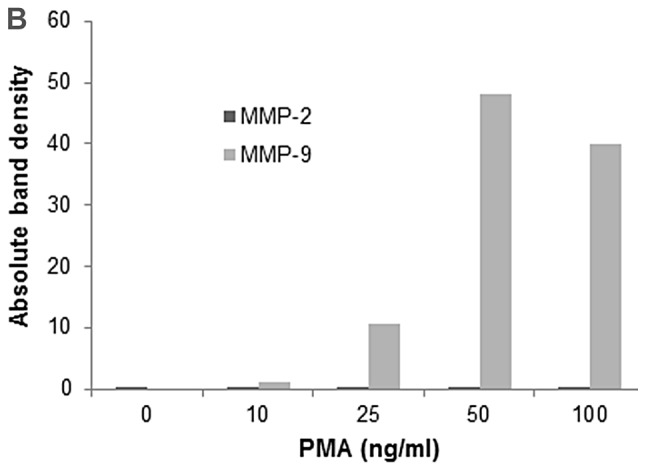
Effect of PMA on rhabdomyosarcoma RD MMP-2 and -9 secretions. Gelatinase zymogram (A) and densitometry analysis (B) of RD MMP-2 and -9 expressions. 1, Markers; 2, control; 3–6, 10, 25, 50 and 100 ng/ml PMA.

On gelatinase zymography, RD cells demonstrated moderate secretion of MMP-9 and imperceptible secretion of MMP-2. PMA treatment strongly stimulated MMP-9 secretion in a dose-dependent manner, with 40.1% at 100 ng/ml, compared to 0% at control (linear trend R^2^=0.794) but had no significant effect on MMP-2, as shown in Fig. 2. TNF-α had a stimulatory dose-dependent effect on MMP-2 (R^2^=0.886) but no effect on MMP-9. IL-1β and LPS were not determined.

Table II shows the quantitative densitometry results from the effects of chemical inhibitors doxycycline dexamethasone, actinomycin-D and cyclohexamide on MMP-2 and -9 secretion by osteosarcoma and rhabdomyosarcoma cell lines.

### Effect of chemical inhibitors doxycycline, dexamethasone, actinomycin-D and cyclohexamide on MMP-2 and -9 secretion in osteosarcoma U2OS cell line

On gelatinase zymography, U2OS cells demonstrated slight secretion of MMP-2 and strong secretion of MMP-9, with enhanced MMP-9 secretion with PMA (100 ng/ml) treatment. Doxycycline with and without PMA (100 ng/ml) treatment showed dose-dependent inhibition of MMP-2 and -9 (linear trends R^2^=0.893 and 0.769, respectively) with 100% inhibition at 100-*μ*M doxycycline for both MMPs compared to control. Actinomycin-D showed dose-dependent inhibition of both MMP-2 and -9 (linear trends R^2^=0.951 and 0.909, respectively), with 96% block of both MMPs at 4 *μ*g/ml. Cyclohexamide showed strong inhibition of both MMPs at dose tested (2 *μ*g/ml). Dexamethasone (100 *μ*M) showed strong inhibition of MMP-2 and -9 compared to control.

### Effect of chemical inhibitors doxycycline, dexamethasone, actinomycin-D and cyclohexamide on MMP-2 and -9 secretion in rhabdomyosarcoma RD cell line

On gelatinase zymography, PMA treatment induced strong secretion of MMP-9 and slight secretion of MMP-2. Doxycycline treatment of PMA-treated (100 ng/ml) RD cells showed dose-dependent inhibition of MMP-9 (linear trend R^2^=0.879), with total block of MMP-9 at 100 *μ*M and MMP-2 at 25 *μ*M. Actinomycin-D showed dose-dependent inhibition of MMP-9 (linear trend R^2^=0.477) with 44% block at 4 *μ*g/ml and no change in MMP-2. Dexamethasone 50 *μ*M demonstrated no effect on MMP-2 but inhibited MMP-9 by 61%. Exposure of RD cells to cyclohexamide was not determined.

Table III shows the quantitative densitometry results from the effects of natural inhibitors EGCG, the NM and retinoic acid on MMP-2 and -9 secretion by osteosarcoma and rhabdomyosarcoma cell lines.

### Effect of natural inhibitors EGCG, the NM and retinoic acid on MMP-2 and -9 secretion in osteosarcoma U2OS cell line treated with PMA

**Figure 3.**
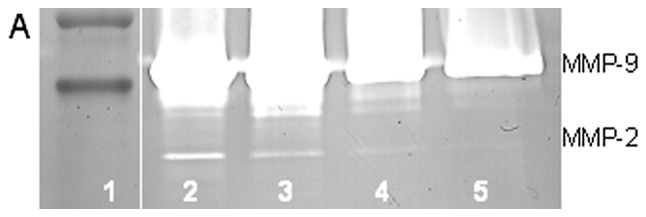

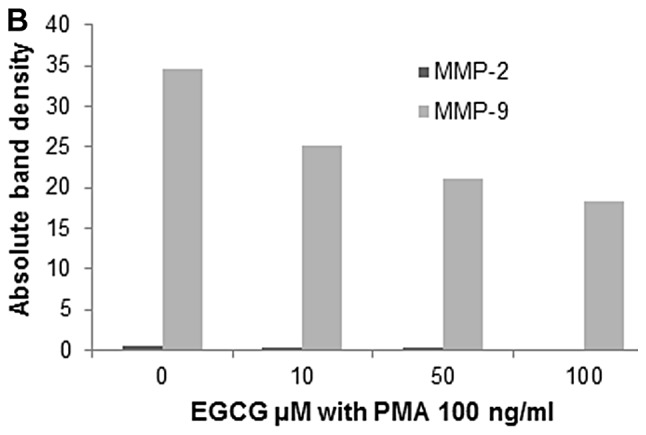
Effect of EGCG on PMA-treated osteosarcoma U2OS MMP-2 and -9 secretions. Gelatinase zymogram (A) and densitometry analysis (B) of U2OS MMP-2 and -9 expressions. 1, Control (100 ng/ml PMA); 2–5, 10, 25, 50 and 100 *μ*M EGCG with PMA (100 ng/ml).

**Figure 4.**
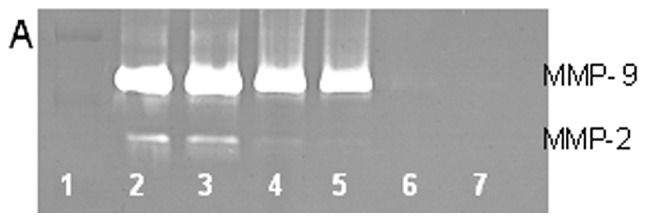

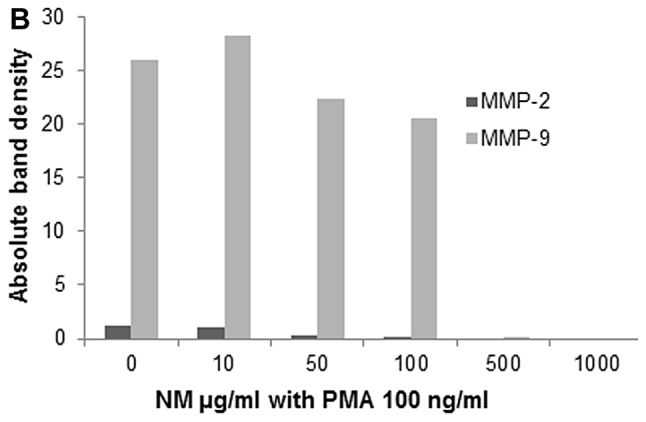
Effect of NM on PMA-treated osteosarcoma U2OS MMP-2 and -9 secretions. Gelatinase zymogram (A) and densitometry analysis (B) of U2OS MMP-2 and -9 expressions. 1, Markers; 2, control (100 ng/ml PMA); 3–7, 10, 50, 100, 500 and 1,000 *μ*g/ml NM with PMA (100 ng/ml).

On gelatinase zymography, U2OS cells demonstrated slight MMP-2 and strong MMP-9 secretion, with enhanced MMP-9 secretion with PMA (100 ng/ml) treatment. In untreated cells, EGCG inhibited MMP-2 by 85% (R^2^=0.903) at 100 *μ*M and MMP-9 by 56% (R^2^=0.674) at 100 *μ*M. In PMA-treated cells, EGCG inhibited MMP-9 and MMP-2 in a dose-dependent manner, with total block of MMP-2 at 100 *μ*M (linear trend R^2^=0.918) and 47% inhibition of MMP-9 at 100 *μ*M (linear trend R^2^=0.870), as shown in Fig. 3. MMPs secreted by untreated cells were inhibited by NM in a dose-dependent manner with total block of MMP-2 and -9 at 500 *μ*g/ml (linear trends R^2^=0.936 and 0.758, respectively). PMA-treated cells showed MMP inhibition by NM in a dose-dependent fashion with total inhibition of MMP-2 at 500 *μ*g/ml (linear trend R^2^=0.840) and MMP-9 at 1,000 *μ*g/ml (linear trend R^2^=0.815). See the gelatinase zymogram and densitometry analysis of NM treatment of PMA-treated U2OS in Fig. 4. Retinoic acid (50 *μ*M) showed strong inhibition of MMP-2 and -9 compared to control.

### Effect of natural inhibitors EGCG, the NM and retinoic acid on MMP-2 and -9 secretion in rhabdomyosarcoma cell line

**Figure 5.**
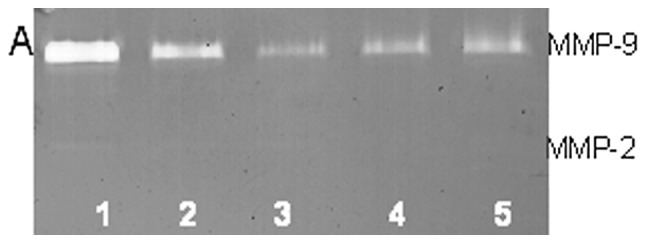

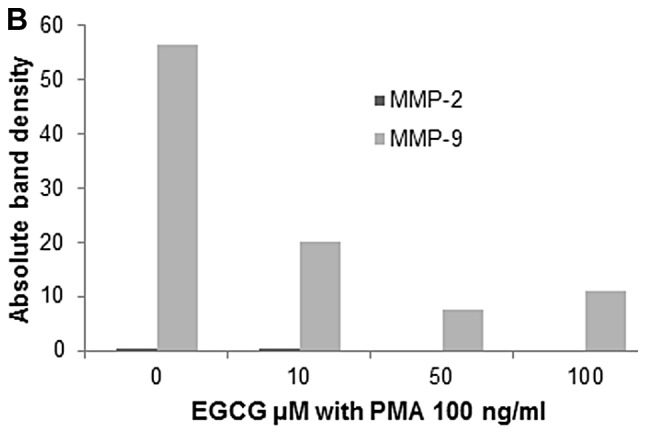
Effect of EGCG on PMA-treated rhabdomyosarcoma RD MMP-2 and -9 secretions. Gelatinase zymogram (A) and densitometry analysis (B) of RD MMP-2 and -9 expressions. 1, Control (100 ng/ml PMA); 2–5, 10, 25, 50 and 100 *μ*M EGCG with PMA (100 ng/ml).

**Figure 6.**
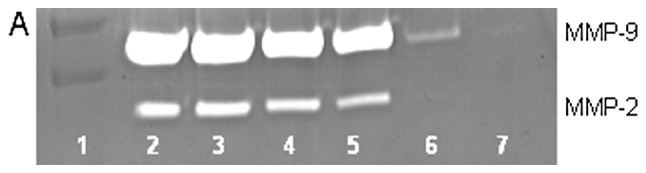

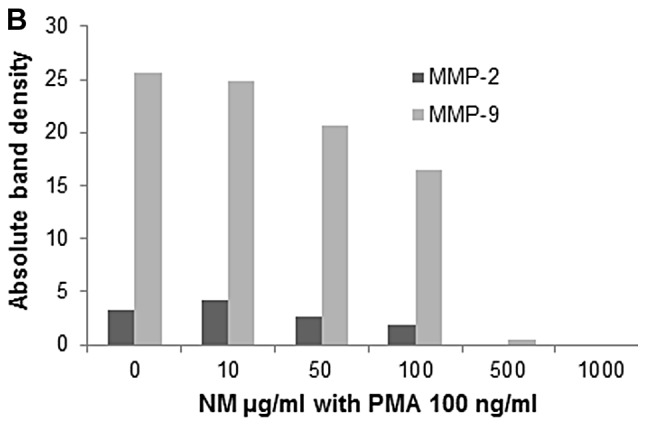
Effect of NM on PMA-treated rhabdomyosarcoma RD MMP-2 and -9 secretions. Gelatinase zymogram (A) and densitometry analysis (B) of RD MMP-2 and -9 expressions. 1, Markers; 2, control (100 ng/ml PMA); 3–7, 10, 50, 100, 500 and 1,000 *μ*g/ml NM with PMA (100 ng/ml).

On gelatinase zymography, untreated RD cells showed strong secretion of MMP-9 and slight secretion of MMP-2. PMA treatment of RD cells profoundly enhanced MMP-9 and decreased MMP-2 secretion. In PMA-treated cells, EGCG inhibited MMP-9 in a dose-dependent manner, with 80.5% inhibition compared to control (linear trend R^2^=0.743), and total block of MMP-2 at 50 *μ*M, as shown in Fig. 5. In untreated RD cells, NM inhibited secretion of MMP-2 and -9 in a dose-dependent manner with total block of MMP-2 at 500 *μ*g/m (linear trend R^2^=0.899) and MMP-9 at 10 *μ*g/ml (linear trend R^2^=0.429). In PMA-treated cells, NM inhibited secretion of MMP-2 and -9 in a dose-dependent manner, with total block of MMP-2 and -9 at 500 *μ*g/ml (linear trends R^2^=0.842 and 0.888, respectively), as shown in Fig. 6. Exposure of RD cells to retinoic acid was not determined.

## Discussion

Tumor aggression and metastasis have been correlated with increased MMP expression (10,11). Overexpression of MMPs, especially MMP-2 and -9 and low levels of TIMPs have been shown to be associated with a more aggressive behavior of sarcomas (8,19–22). For example, increased expression of MMP-9 has been found to correlate with osteosarcoma metastasis in patients and inhibitors of MMPs, such as TIMP-1 have been shown to inhibit invasiveness of osteosarcoma tumor cells *in vitro* (19–21). A study of the immunohistochemical expression of MMPs and TIMPS in human rhabsomyosarcoma revealed strong MMP-1, -3 and -9 expression in rhabdomyosarcoma, alveolar RMS greater than embryonal RMS. Intratumor vessels and perivascular ECM were positive for MMP-9 and negative for TIMPS in both types (23).

Thus, knowledge of MMP regulation is of importance for developing therapeutic strategies. MMP expression is regulated at both pre- and post-transcriptional levels. Extracellular factors, including cytokines, growth factors, and inducers and inhibitors, have been implicated in the regulation of MMP expression in different types of tumor cells (24,25). Though few cytokine and growth factor studies have been conducted on sarcomas, some research has documented elevated serum levels of VEGF, IL-2 and bFGF in sera of patients with soft tissue sarcomas (26,27); VEGF serum levels correlated significantly with tumor size and histological grade (26). Serum cytokine levels significantly correlated with tumor size and grade suggesting involvement of cytokines in the progression of soft tissue sarcomas (18). Rutkowski *et al* found elevated cytokines and soluble cytokine receptors involved in bone destruction and bone formation in 46% of adult bone sarcoma patients, suggesting they have an essential role in the progression of malignant bone tumors (28).

In this study, we compared MMP secretion patterns by cytokines, PMA, and LPS in two pediatric sarcoma cell lines that express MMP-2 and -9 to different extent. In addition, we investigated the effect of inhibitors doxycycline and EGCG and others, such as dexamethasone, retinoic acid and agents that affect transcription and translation levels, such as actinomycin-D and cyclohexamide. Furthermore, we tested a nutrition mixture that had inhibitory effects on MMP-2 and -9 secretion. We found that osteosarcoma U2OS and rhabdomyosarcoma RD normally secreted both MMP-2 and -9. Treatment of these cell lines with PMA strongly upregulated secretion of MMP-9 in a dose-dependent manner but had no effect on secretion of MMP-2. TNF-α had a stimulatory effect on U2OS secretion of MMP-9 but no effect on MMP-2, while it stimulated MMP-2 secretion in RD cells, but had no effect on MMP-9. IL-1β and LPS stimulated MMP-9 in osteosarcoma U2OS cells, but no significant effect on MMP-2.

Doxycycline and EGCG inhibited MMP-2 and -9 secretion in a dose-dependent fashion in both cell lines tested. Sensitivity to doxycycline and EGCG in normal osteosarcoma U2OS cells was nearly equivalent in MMP-2 secretion, but secretion of MMP-9 was significantly more sensitive to doxycycline than EGCG. In PMA-treated U2OS cells and RD cells, there was very slight MMP-2 secretion; however, MMP-9 was strongly enhanced. Doxycycline completely blocked MMP-9 at 100 *μ*M in both cell lines, whereas 100 *μ*M EGCG down-regulated MMP-9 in U2OS by 47% and in RD cells by 81%. The nutrition mixture inhibited MMP-2 and -9 secretion in a dose-dependent fashion in normal and PMA-treated U2OS and RD cells. Sensitivity of normal U2OS and RD cells to NM were similar with respect to MMP-2 secretion with total block of MMP-2 at 500 *μ*g/ml; however, MMP-9 was blocked in U2OS cells at 1,000 *μ*g/ml and in RD cells at 10 *μ*g/ml. Sensitivity of PMA-treated U2OS and RD cells were equally sensitive to NM with total block of MMP-2 at 500 *μ*g/ml and MMP-9 at 1,000 *μ*g/ml. Actinomycin-D, cyclohexamide, retinoic acid, and dexamethasone inhibited MMP-2 and -9 in osteosarcoma cells. In RD cells, dexamethasone and actinomycin-D showed inhibition of MMP-9 secretion.

The nutrition mixture studied, which contains lysine, proline, ascorbic acid, and green tea extract among other micro-nutrients, has been shown to have antitumor and anti-invasive potential *in vivo* and *in vitro* (29). Of interest, a previous study demonstrated significant correlation between NM inhibition of Matrigel invasion and NM modulation of the MMP-2 and -9 activities of the sarcoma cell lines studied (30). A significant negative correlation was found between NM modulation of Matrigel invasion inhibition and MMP-2 secretion with osteosarcoma U2OS (r=−0.835) and rhabdomyosarcoma RD (r=−0.675).

The nutrient mixture was formulated by selecting nutrients that act on critical physiological targets in cancer progression and metastasis, as documented in both clinical and experimental studies. Combining these micronutrients expands metabolic targets, maximizing biological impact with lower doses of components. A previous study of the comparative effects of NM, green tea extract and EGCG on inhibition of MMP-2 and -9 secretion of different cancer cell lines with varying MMP secretion patterns, revealed the superior potency of NM over GTE and EGCG at equivalent doses (31). These results can be understood from the more comprehensive treatment offered by the combination of nutrients in NM over individual components of NM since MMP-2 and -9 are mediated by differential pathways.

Optimal ECM structure depends upon adequate supplies of ascorbic acid and the amino acids lysine and proline to ensure proper synthesis and hydroxylation of collagen fibers. In addition, lysine contributes to ECM stability as a natural inhibitor of plasmin-induced proteolysis (32,33). Manganese and copper are also essential for collagen formation. There is considerable documentation of the potency of green tea extract in modulating cancer cell growth, metastasis, angiogenesis, and other aspects of cancer progression (34–38). N-acetyl cysteine and selenium have demonstrated inhibition of tumor cell MMP-9 and invasive activities, as well as migration of endothelial cells through ECM (39–41). Ascorbic acid demonstrates cytotoxic and antimetastatic actions on malignant cell lines (42–46) and cancer patients have been found to have low levels of ascorbic acid (47,48). Low levels of arginine, a precursor of nitric oxide (NO), can limit the production of NO, which has been shown to predominantly act as an inducer of apoptosis (49).

In conclusion, our results show that cytokines, inducers and inhibitors regulate MMP-2 and -9 secretion in pediatric sarcoma cell lines, suggesting the clinical value of targeting these proteases for management of sarcomas and their pathogenesis.

## Figures and Tables

**Table I.** Effect of inducers on pediatric sarcoma MMPs.

	Osteosarcoma (U2OS)		Rhabdomyosarcoma (RD)	
	MMP-2	MMP-9	MMP-2	MMP-9
PMA (ng/ml)				
Control	0.4%	2.5%	0.0%	0.0%
10	0.3%	11.4%	0.0%	1.1%
25	0.3%	28.2%	0.0%	10.7%
50	0.3%	26.3%	0.0%	48.1%
100	0.3%	30.0%	0.0%	40.1%
TNF-α (ng/ml)				
Control	1.0%	8.8%	0.0%	0.0%
0.1	1.4%	13.9%	3.3%	0.0%
1	1.0%	14.6%	3.7%	0.0%
10	1.0%	23.7%	8.0%	0.0%
25	0.9%	33.8%	15.9%	0.0%
IL-1β (ng/ml)				
Control	1.1%	8.8%	ND	ND
0.1	1.0%	12.7%	ND	ND
1	1.0%	25.0%	ND	ND
10	1.1%	23.5%	ND	ND
25	0.6%	25.2%	ND	ND
LPS (*μ*g/ml)				
Control	1.6%	9.7%	ND	ND
10	1.9%	22.4%	ND	ND
25	1.7%	20.6%	ND	ND
50	0.9%	22.3%	ND	ND
100	1.1%	18.0%	ND	ND

**Table II.** Effect of chemical inhibitors on pediatric sarcoma MMPs.

	Osteo-sarcoma (U2OS)		Rhabdomyosarcoma (RD)	
	MMP-2	MMP-9	MMP-2	MMP-9
Doxycycline (*μ*M)				
Control	1.9%	20.2%	ND	ND
10	2.3%	20.6%	ND	ND
25	0.5%	25.7%	ND	ND
50	0.4%	25.0%	ND	ND
100	0.0%	3.4%	ND	ND
Doxycycline (*μ*M) with PMA (100 ng/ml)				
Control	0.9%	26.1%	0.4%	46.6%
10	1.0%	32.5%	0.1%	20.2%
25	0.6%	23.3%	0.0%	23.0%
50	0.1%	15.6%	0.0%	9.7%
100	0.0%	0.0%	0.0%	0.0%
Dexamethasone (*μ*M)				
Control	17.8%	69.4%	0.0%	72.0%
50	4.4%	8.4%	0.0%	28.0%
Actinomycin-D (*μ*g/ml)				
Control	15.5%	60.4%	0.0%	51.4%
2	5.1%	15.7%	0.0%	19.7%
4	0.6%	2.6%	0.0%	28.9%
Cyclohexamide (*μ*g/ml)				
Control	20.1%	78.3%	ND	ND
2	0.3%	1.3%	ND	ND

**Table III.** Effect of natural inhibitors on pediatric sarcoma MMPs.

	Osteo-sarcoma (U2OS)		Rhabdomyosarcoma (RD)	
	MMP-2	MMP-9	MMP-2	MMP-9
EGCG (*μ*M)				
Control	2.7%	22.9%	ND	ND
10	1.4%	33.3%	ND	ND
50	0.7%	24.0%	ND	ND
100	0.4%	10.1%	ND	ND
EGCG (*μ*M) with PMA (100 ng/ml)				
Control	0.6%	34.5%	0.3%	56.5%
10	0.2%	25.1%	0.1%	20.0%
50	0.1%	21.0%	0.0%	7.6%
100	0.0%	18.4%	0.0%	11.0%
Nutrient mixture (*μ*g/ml)				
Control	30.2%	2.46%	28.7%	3.3%
10	30.9%	2.24%	29.9%	0.0%
50	20.0%	0.62%	21.0%	0.0%
100	11.9%	1.66%	17.1%	0.0%
500	0.0%	0.0%	0.0%	0.0%
1,000	0.0%	0.0%	0.0%	0.0%
Nutrient mixture (*μ*g/ml) with PMA (100 ng/ml)				
Control	1.2%	26.0%	3.2%	25.7%
10	1.1%	28.2%	4.2%	24.9%
50	0.3%	22.3%	2.6%	20.7%
100	0.1%	20.6%	1.8%	16.5%
500	0.0%	0.1%	0.0%	0.5%
1,000	0.0%	0.0%	0.0%	0.0%
Retinoic acid (*μ*M)				
Control	14.3%	55.7%	ND	ND
50	5.2%	24.9%	ND	ND
